# Bridging linguistic reasoning and biophysical reality toward peptide engineering via instruction-tuned language modeling

**DOI:** 10.1093/bioinformatics/btag618

**Published:** 2026-08-17

**Authors:** Kaijun Yang, Tianxiang Wu, Wenbo Zhang, Pengyong Li

**Affiliations:** School of Computer Science and Technology, Xidian University, Xi’an, Shaanxi 710126, China; School of Computer Science and Technology, Xidian University, Xi’an, Shaanxi 710126, China; School of Computer Science and Technology, Xidian University, Xi’an, Shaanxi 710126, China; School of Computer Science and Technology, Xidian University, Xi’an, Shaanxi 710126, China

## Abstract

**Motivation:**

Peptides serve as critical mediators in biological systems, regulating essential processes ranging from neurotransmission to immune response. However, their nonlinear sequence–function relationships and immense chemical diversity pose significant challenges for efficient experimental characterization and therapeutic development. While Protein Language Models have advanced biological sequence understanding, they predominantly capture global evolutionary features of full-length proteins, often overlooking the local physicochemical dependencies and short-range residue interactions that are essential for defining peptide bioactivity.

**Results:**

To address this gap, we leverage the linguistic competence of general-purpose Large Language Models (LLMs) to treat amino acid sequences as “biological text,” bridging natural language supervision with biochemical sequence modeling without relying on explicit structural or evolutionary priors. We curate a peptide-specific instruction dataset, Pep-Instructions, spanning function description, sequence design, property prediction, and physicochemical optimization, and adapt a general-purpose LLM through parameter-efficient instruction tuning. Extensive benchmarking against general-purpose language models shows consistent improvements across the evaluated peptide-centric tasks. In particular, the instruction-tuned model produces more semantically faithful functional descriptions, generates peptide sequences with stronger sequence-level similarity, with representative ESMFold case studies suggesting backbone-level consistency, improves prediction of diverse peptide properties, and enables more reliable directional optimization of physicochemical properties under the adopted in silico evaluation protocols. Overall, these results establish Pep-Instructions as a unified benchmark and demonstrate the value of peptide-specific instruction tuning for peptide understanding, prediction, and design.

**Availability and implementation:**

Source code and Pep-Instructions are available at https://github.com/kjY7836/pepinstruction. Fine-tuned model weights are available at https://huggingface.co/Codelife176/Pep-instruction.

## 1 Introduction

Peptides, composed of short chains of amino acids, play crucial roles in biological systems as hormones, antimicrobial agents, signaling molecules, and immune regulators. Owing to their structural simplicity, high specificity, and tunable physicochemical properties, peptides have emerged as promising candidates for therapeutic design and biomaterial engineering (Muttenthaler *et al.* 2021). However, the nonlinear and context-dependent relationships between peptide sequence and biological function make experimental characterization of their activity and stability both time-consuming and expensive. Consequently, computational approaches capable of modeling the complex mapping between sequence and function have become indispensable for accelerating peptide discovery and optimization (Das *et al.* 2021, Zhang *et al.* 2023).

Recent years have witnessed a paradigm shift driven by the emergence of *Large Language Models* (LLMs) (Brown *et al.* 2020, Ouyang *et al.* 2022), which have demonstrated remarkable capability in capturing contextual dependencies from large-scale unlabeled data through self-supervised pretraining. Since amino acid sequences can be represented as symbolic strings governed by contextual grammar and semantics, similar to human language (Ofer *et al.* 2021), the language modeling paradigm has been successfully extended to biological macromolecules (Rives *et al.* 2021), giving rise to *Protein Language Models* (PLMs) (Jumper *et al.* 2021, Madani *et al.* 2023). Representative PLMs such as ESM (Lin *et al.* 2023) and ProtBERT (Elnaggar *et al.* 2021) have achieved state-of-the-art performance in protein structure prediction, function annotation, and mutation effect estimation, confirming the effectiveness of language modeling as a sequence-only paradigm for understanding biological macromolecules. Extending this to generative tasks, LM-Design (Zheng *et al.* 2023) showed that PLMs can enable structure-based sequence design via lightweight structural adapters, advancing structure-conditioned protein engineering.

Building upon this foundation, researchers have begun to explore whether general-purpose Large Language Models (Luo *et al.* 2022, Fang *et al.* 2024)—pretrained on massive natural language corpora—can leverage their strong contextual reasoning and generative capabilities to model biological sequences (Hayes *et al.* 2025). By treating amino acid sequences as a form of “biological text,” these models offer a new paradigm that complements traditional PLMs. Notable advancements have focused on adapting the LLaMA (Touvron *et al.* 2023) architecture for biology. For instance, ProLLaMA (Lv *et al.* 2026) shows that a general-purpose LLaMA model can be transformed into a multi-task protein language model through vocabulary pruning and instruction tuning. In parallel, the Open Protein Instruction (OPI) dataset (Xiao *et al*. 2024) provides large-scale instruction-tuning data that aligns LLaMA-based architectures with protein understanding and design objectives. Collectively, these developments highlight the strong potential of leveraging natural language models as universal sequence learners across biological domains.

Despite these promising advances, both PLMs and LLM-derived protein models remain limited when applied to peptide-level tasks (Guntuboina *et al.* 2023). Their pretraining objectives are predominantly centered on full-length proteins, biasing representations toward global evolutionary patterns and overall fold-level organization—factors less relevant to peptides. As a result, these models struggle to capture short-range residue interactions, local physicochemical dependencies, and fine-grained sequence patterns that underlie key peptide properties such as solubility, hydrophobicity, and bioactivity (Szymczak *et al.* 2023). Moreover, peptide sequences exhibit distinct statistical characteristics due to their shorter length and functional diversity, causing a mismatch with protein-centric training distributions. Effective adaptation for peptide property prediction and sequence design therefore requires task-oriented fine-tuning. However, peptide datasets are typically small and noisy, making full-parameter fine-tuning computationally expensive and prone to catastrophic forgetting (Luo *et al.* 2025). These challenges underscore the need for Parameter-Efficient Fine-Tuning (PEFT) methods such as Low-Rank Adaptation (LoRA) (Hu *et al.* 2022, Wang *et al.* 2024, Zeng *et al.* 2024), which introduce small trainable low-rank updates into frozen weight matrices, achieving domain adaptation with minimal parameter overhead while preserving the generalization capacity obtained during pretraining.

To address these limitations, we develop a peptide-centred instruction-tuning framework based on a newly curated benchmark and parameter-efficient adaptation of a general-purpose large language model. Specifically, this work makes three contributions:

- **Data level.** We construct *Pep-Instructions*, a peptide-specific instruction dataset containing 482 639 instruction–response pairs spanning four task categories. We further apply unified sequence-level splitting with exact canonical-sequence deduplication across all sequence lengths and length-aware similarity control for sequence pairs with $L_{\text{min}}\geq 8$, supporting a more reliable evaluation setting.
- **Task level.** We unify *function description*, *sequence design*, *property prediction*, and *physicochemical optimization* within a single benchmark, enabling joint assessment of both discriminative and generative peptide modeling capabilities under a consistent evaluation setting.
- **Method level.** We show that LoRA-based instruction tuning of *LLaMA2-7B*, with adaptation applied to both attention and feed-forward components, can consistently improve performance over general-purpose baselines on multiple peptide-centric tasks using raw peptide sequences alone, without explicit structural or evolutionary priors.

## 2 Experimental

### 2.1 Overall framework

**Figure 1:**
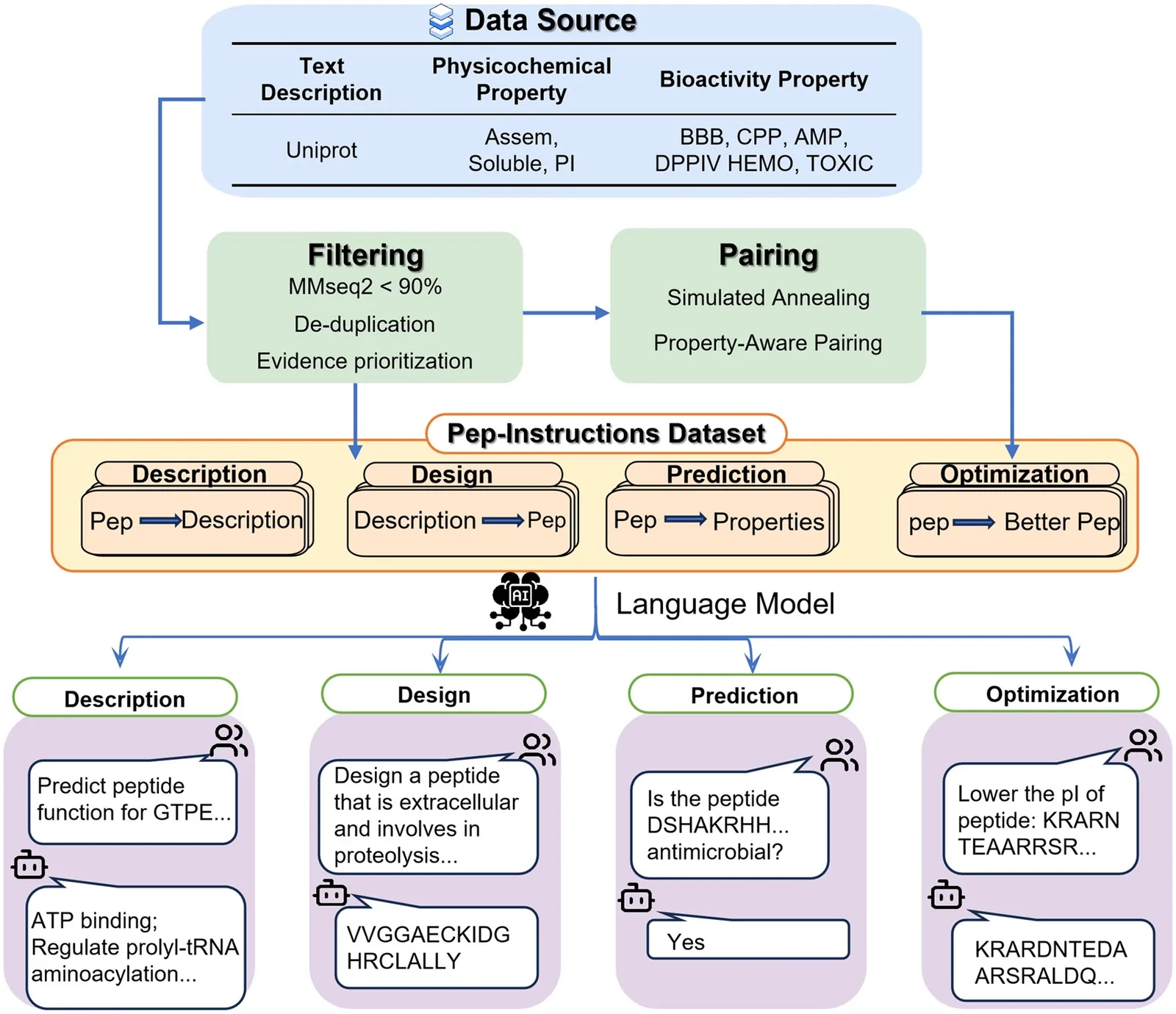
Overall framework of Pep-Instructions for peptide instruction tuning. The upper panel illustrates the benchmark construction pipeline, including data curation, task formulation, and dataset organization. The lower panel summarizes the four task categories supported in Pep-Instructions, namely function description, sequence design, property prediction, and optimization, together with representative instruction–response examples.

In this study, we develop a unified instruction-tuning framework that adapts a general-purpose large language model into a peptide-specialized language model for understanding, reasoning about, and generating peptides (Fig. 1). The overall framework consists of three stages: (i) constructing **Pep-Instructions**, a large-scale peptide instruction dataset covering diverse peptide-related tasks, (ii) fine-tuning LLaMA2-7B with Low-Rank Adaptation to align natural-language instructions with peptide sequence modeling, and (iii) systematically evaluating the adapted model across multiple discriminative and generative settings.

Pep-Instructions comprises **4 82 639 peptide–instruction pairs** spanning four representative task categories: *Function Description* (1 03 036), *Sequence Design* (1 05 525), *Property Prediction* (84 748), and *Optimization* (1 89 330). These tasks jointly cover peptide interpretation, conditional generation, attribute inference, and controllable sequence refinement, thereby providing a unified benchmark for instruction tuning and downstream evaluation in peptide modeling.

### 2.2 Experimental design

The experimental work primarily consists of three major components: the construction of a task–instruction dataset, the instruction fine-tuning of the LLaMA2-7B model, and the comprehensive evaluation of its performance across multiple peptide-related tasks.

### 2.3 Dataset construction

#### 2.3.1 Data sources and preprocessing

The dataset is derived from two main sources: *UniProt* and our curated database. Regarding the data sourced from *UniProt*, we first extract polypeptide sequences with lengths ranging from 1 to 50 amino acids, and then employ *MMseqs2* (Many-against-Many sequence searching 2) (Steinegger and Söding 2017) to filter and cluster these sequences. *MMseqs2* is a fast sequence-alignment and homology-searching tool that identifies sequences similar to the target polypeptides from large-scale databases, constructs multiple sequence alignments, and performs clustering and redundancy removal to ensure dataset diversity and high quality. For the initial *UniProt*-derived sequences, we set a sequence-similarity threshold below 90% to remove redundant entries. The resulting diverse set of polypeptides serves as the foundation for the *function description* and *design* tasks.

#### 2.3.2 Construction of function description and sequence design tasks

The *function description* task infers biological roles from polypeptide sequences using the *Gene Ontology* (GO) system. This involves terms across three standardized categories: *Biological Process* (BP), *Molecular Function* (MF), and *Cellular Component* (CC), which respectively describe the peptide’s associated processes, activities, and localization. Based on the associated Gene Ontology terms, we filtered out non-interpretable information and extracted task-relevant functional annotations, which were subsequently reformulated into concise natural language descriptions to characterize the biological functions of the corresponding polypeptides.

The *design* task is formulated to generate polypeptide sequences that satisfy predefined functional and structural constraints. These constraints are derived from manually curated *UniProt* metadata, including *Function*, *GO annotation*, *Catalytic activity*, *Domain*, *Cofactor*, *Pathway*, *Motif*, *Signal peptide*, *DNA binding*, and *Transmembrane* features, following the structured scientific instruction generation process illustrated in Fig. S1, available as supplementary data at *Bioinformatics* online. For each instruction instance, we included at most six non-empty attributes from the biologically curated attribute pool. When more than six attributes were available in the selected source record, six were randomly sampled (Table S1, available as supplementary data at *Bioinformatics* online). Thus, six represents a prespecified upper bound on instruction complexity rather than a fixed or empirically optimized attribute count.

#### 2.3.3 Construction of property prediction and physicochemical optimization tasks

The *property prediction* and *optimization* tasks were established based on data curated from our proprietary peptide database. Specifically, the *property prediction* task encompasses eight functional and physicochemical property types, including *Assem* (self-assembling peptides), *Amp* (antimicrobial peptides), *BBB* (blood–brain–barrier–penetrating peptides), *CPP* (cell-penetrating peptides), *DPPIV* (DPP-IV inhibitory peptides), *Hemo* (hemolytic peptides), *Soluble* (solubility-associated peptides), and *Toxic* (toxic peptides).

For peptides obtained from the curated database, we first computed key physicochemical properties—including isoelectric point, net charge, LogP, and instability index—using the *Peptidy* (Özçelik *et al.* 2025) Python toolkit, which is designed for peptide sequence preprocessing in machine-learning workflows. These descriptors are central to peptide drug discovery and stability optimization, as they capture properties relevant to biological activity and *in vivo* behavior. Building on these features, we formulated the *optimization* task by applying a Simulated Annealing (SA) strategy to generate property-improved peptide variants while preserving high sequence similarity to the original inputs (similarity score >0.8). For each property-oriented objective, all physicochemical attributes—except for the instability index, which was consistently reduced—were perturbed in both increasing and decreasing directions, yielding an approximately balanced set of optimization instructions. Representative instruction-response pairs for four tasks are visualized in Fig. S2, available as supplementary data at *Bioinformatics* online.

### 2.4 Unified sequence-level splitting and similarity control

To prevent benchmark leakage and performance inflation, we re-partitioned Pep-Instructions at the sequence level using a unified protocol of exact-duplicate removal and homology-aware clustering. For function description and property prediction, the canonical peptide sequence was extracted from the input field; for sequence design, it was extracted from the output sequence; and for optimization, both input and output sequences were included. To prevent information transfer through paired optimization examples, optimization input-output pairs were linked and treated as indivisible units during splitting.

We further performed exact deduplication across all tasks so that the same peptide sequence, even if associated with different instructions or task formulations, could not appear in multiple splits. To reduce homology-based leakage, we constructed sequence-level homology graphs using MMseqs2 all-versus-all search followed by a length-aware thresholding rule. For a candidate sequence pair, we define L_min=min(L_i,L_j) as the length of the shorter sequence. Non-exact similarity edges were retained when one of the following conditions was satisfied: (i) identity ≥0.8 and alignment length ≥8 for 8≤L_min≤10, (ii) identity ≥0.7 and alignment length ≥10 for 11≤L_min≤20, or (iii) identity ≥0.5 and coverage ≥0.8 for L_min>20. For L_min<8, no non-exact similarity edges were introduced; these sequences were controlled through exact canonical-sequence deduplication across splits.

These thresholds are operational benchmark-construction criteria rather than universal definitions of peptide homology. For short peptides, percentage identity is highly discrete and short matches can occur by chance; requiring a minimum absolute alignment length reduces weak motif-driven edges and transitive chaining. For longer peptides, the identity threshold is relaxed while high coverage is required to capture sequence-wide rather than local similarity.

Connected components induced by these exact and homologous links were used as the minimum split units. Based on these components, a single task-agnostic global split with a ratio of 8:1:1 was performed for training, validation, and testing. Finally, we applied an iterative cross-split homology audit-and-repair procedure until zero exact canonical-sequence overlap existed for all lengths, and no cross-split pair with $L_{\text{min}}\geq 8$ met our length-dependent similarity thresholds. For property prediction, minority-class samples were randomly oversampled by simple replication within each property-specific training subset to achieve an approximately balanced class distribution; the validation and test subsets were kept unchanged, and the global multi-task split was not altered. Endpoint and split-specific positive and negative counts, positive-class prevalence, and the effective training composition before and after oversampling are reported in Table S2, available as supplementary data at *Bioinformatics* online.

### 2.5 Model fine-tuning

During instruction fine-tuning, parameter-efficient adaptation was performed on the pretrained LLaMA-2-7B model. Training was conducted on a custom instruction dataset using two NVIDIA RTX 5090 GPUs. A per-device batch size of 32 with four-step gradient accumulation resulted in an effective batch size of 256, and the model was trained for seven epochs. To improve numerical stability and memory efficiency, bfloat16 (BF16) mixed-precision training and gradient checkpointing were employed. FlashAttention (Shah *et al.* 2024) was enabled to accelerate attention computation. Optimization used AdamW with an initial learning rate of $2\times 10^{-4}$, no weight decay, and a maximum gradient norm of 0.3. A linear warm-up over 5% of total steps was followed by cosine decay, yielding the convergence profile shown in Fig. S3, available as supplementary data at *Bioinformatics* online.

To enable efficient task-specific adaptation, the LoRA framework was adopted. LoRA injects trainable low-rank matrices into selected linear layers while keeping pretrained weights frozen. Each weight matrix $W\in R^{d\times k}$ is reparameterized as


(1) $$W^{\prime }=W+\alpha BA,$$

where $A\in R^{r\times k}$ and $B\in R^{d\times r}$ are learnable matrices, *r* is the rank, and α is a scaling factor. In this work, r=64, α=128, and dropout is set to 0.1.

Beyond conventional settings that restrict LoRA to attention sublayers, we extend adaptation to both attention and feed-forward network (FFN) components. Specifically, LoRA is applied to $q_{\text{proj}},k_{\text{proj}},v_{\text{proj}},o_{\text{proj}}$ in attention blocks, as well as *gate_proj*, *up_proj*, and *down_proj* in FFN blocks. This design follows recent findings that adapting all linear layers significantly narrows the gap with full fine-tuning (Dettmers *et al.* 2023).

This strategy leverages the complementary roles of Transformer substructures: attention models cross-token dependencies, while FFN layers capture factual knowledge and perform channel mixing (Geva *et al.* 2021, Dai *et al.* 2022). Joint LoRA adaptation across both components improves inter-token and intra-token representations, enhancing contextual reasoning, generation quality, and task adaptability without substantial computational overhead.

### 2.6 Evaluation protocol and metrics

The evaluation consisted of two levels. We first characterized the full Pep-Instructions corpus in terms of sequence diversity and split integrity. We then evaluated model performance on the held-out test set across four downstream tasks: function description, sequence design, property prediction, and physicochemical optimization.

#### 2.6.1 Corpus-level diversity characterization and split validation

We evaluated the Pep-Instructions corpus at two levels: overall sequence diversity and split integrity. For corpus-level characterization, amino acid sequences from all four task categories were standardized to contain only canonical residues (A–Y). Amino acid frequencies were aggregated over the full dataset, and Shannon entropy ($H_{\text{Shannon}}$) was computed from the normalized residue distribution to quantify compositional diversity.

To further assess diversity in representation space, each standardized sequence was encoded using pretrained ProtBERT embeddings. The global structure of the embedding space was visualized using Uniform Manifold Approximation and Projection (UMAP) projection, providing a qualitative view of sample dispersion and inter-task distribution patterns. To quantify distributional differences across task categories, pairwise Kullback–Leibler (KL) divergences were computed between Gaussian approximations of the embedding distributions reduced by principal component analysis (PCA):


(2) $$\begin{array}{cc} D_{\text{KL}}(P∥Q)=\frac{1}{2}[ & \text{tr}\left(\Sigma_{Q}^{-1}\Sigma_{P}\right)+{\left(\mu_{Q}-\mu_{P}\right)}^{⊤}\Sigma_{Q}^{-1}(\mu_{Q}-\mu_{P})\\ & -k+\text{ln}\frac{\text{det}\Sigma_{Q}}{\text{det}\Sigma_{P}}], \end{array}$$

where $\mu_{P}$, $\mu_{Q}$ and $\Sigma_{P}$, $\Sigma_{Q}$ denote the means and covariance matrices of the two Gaussian distributions, and *k* is the dimensionality of the PCA-reduced space.

Together, these analyses provide a compact quantitative description of corpus diversity and task-level representational separability, supporting subsequent evaluation of benchmark coverage and split quality.

#### 2.6.2 Function description evaluation

To ensure a fair and systematic comparison of the generated functional descriptions, we adopted a multi-level evaluation framework encompassing both lexical fidelity and semantic alignment.

First, to quantify surface-level textual reconstruction, we employed **BLEU**, *ROUGE-L*, and keyword-level *Exact Match*. *BLEU* measures n-gram precision and *ROUGE-L* captures sentence-level structural overlap through the longest common subsequence. Keyword-level *Exact Match* is defined as a strict exact match between the set of critical Gene Ontology terms in the prediction and that in the reference, thereby assessing the precise recovery of standardized biological nomenclature.

Complementing these lexical measures, we incorporated *Semantic Metrics* using *BERTScore* (Zhang *et al.* 2019) (based on roberta-large) to evaluate the biological validity of the descriptions beyond literal overlap. Unlike rigid keyword matching, BERTScore leverages contextual embeddings to compute soft similarity, allowing for the recognition of synonymous biological expressions and distinct phrasing that convey equivalent functional meaning. Taken together, this dual-metric strategy ensures that our evaluation captures both the precise terminological adherence (Lexical) and the underlying biological correctness (Semantic) of the model’s predictions.

#### 2.6.3 Design task evaluation

For the *design* task, model performance was evaluated by measuring the sequence-level similarity between the generated peptides and their corresponding reference targets. Two complementary alignment-based similarity measures were employed to assess the character-level and biochemical fidelity of the generated sequences.

First, a global character-level alignment was used to compute a normalized sequence identity score that quantifies the positional overlap between predicted and target residues. While this metric provides an intuitive assessment of literal sequence similarity, it does not fully capture biochemical equivalence among amino acids with comparable physicochemical properties. To address this limitation, we further introduced a biologically informed similarity metric based on the BLOSUM80 substitution matrix, which offers higher sensitivity for closely related short peptides (<50 amino acids). Each generated–target pair was globally aligned under a BLOSUM80 scoring scheme with softened gap penalties (gap open = −4, extend = −0.1), and the resulting scores were normalized through a self-comparison scheme, where the raw alignment score was divided by the mean of self-alignment scores from both sequences. This normalization ensures score stability and comparability across sequences of varying lengths and residue compositions. This biologically grounded similarity assessment thus provides a more functionally meaningful measure than pure lexical matching, allowing for a nuanced evaluation of the model’s capability to preserve biochemical motifs and physicochemical constraints during peptide design.

#### 2.6.4 Property prediction evaluation

For the *property prediction* task, our evaluation protocol is designed to assess the model’s capability to infer functional bioactivities directly from amino acid sequences. Given that the language model generates textual responses (e.g. “Yes” or “No”), we map these outputs to discrete binary labels. While prediction accuracy provides a straightforward measure of alignment between generated answers and ground-truth annotations, it is intrinsically sensitive to class imbalance. The direction and magnitude of this imbalance vary substantially across the eight endpoints, as detailed in Table S2, available as supplementary data at *Bioinformatics* online. In such skewed distributions, high accuracy can be misleadingly attained by biasing predictions toward the majority class, thereby masking the model’s inability to identify specific functional traits.

To mitigate these biases and ensure a rigorous assessment, we adopt the Macro-F1 score as our primary metric. Calculated as the unweighted arithmetic mean of the per-class F1 scores, Macro-F1 treats both the minority and majority classes with equal importance, independent of their sample support. Unlike accuracy or micro-averaged metrics, Macro-F1 prevents the dominant class from overshadowing the model’s performance on the scarce class. Consequently, this metric provides a more holistic view of the model’s discriminatory power, ensuring that the reported performance reflects a genuine generalization capability across all functional categories rather than statistical artifacts of the dataset distribution.

#### 2.6.5 Physicochemical optimization task evaluation

To rigorously assess the model performance, we quantified the physicochemical shifts between input and output sequences using the *Peptidy* Python library. The evaluation framework focused on whether generated sequences achieved the requested directional property shift across four physicochemical properties, specifically: Stability Optimization through the minimization (↓) of the instability index to enhance structural stability; Electrostatic Modulation via the bidirectional optimization (↑ and ↓) of the isoelectric point (pI) and net charge, facilitating precise control over pH response and ionic interactions; and Energetic Profiling, involving the directional regulation (↑ and ↓) of the LogP descriptor to modulate lipophilic potential.

For each optimization trial, success was determined by verifying whether the numerical deviation of the target property aligned with the pre-defined optimization vector. Consequently, the model’s performance was quantified by the Success Rate, defined as the proportion of generated sequences that successfully satisfied these directional constraints relative to the total number of evaluation samples.

To assess evaluation-time stability, sequence design was repeated with three decoding seeds and reported as mean ± standard deviation. Function description, property prediction, and physicochemical optimization used greedy decoding for our model and the baselines, yielding identical outputs across three repeated runs. For *GPT-3.5-Turbo*, the main benchmark tables report results from the primary recorded API run. Repeatability was additionally assessed using three independent API calls at temperature 0 and is summarized separately as mean ± standard deviation in Table S3, available as supplementary data at *Bioinformatics* online. Finite-test-set uncertainty was further estimated using 2000 bootstrap resamples with 95% percentile confidence intervals. Resampling was class-stratified for property prediction and performed at the source-peptide level for the other two tasks; paired bootstrap comparisons with the strongest baseline are reported in Table S3, available as supplementary data at *Bioinformatics* online.

## 3 Results

### 3.1 Statistical profile and split validation of Pep-Instructions

**Figure 2:**
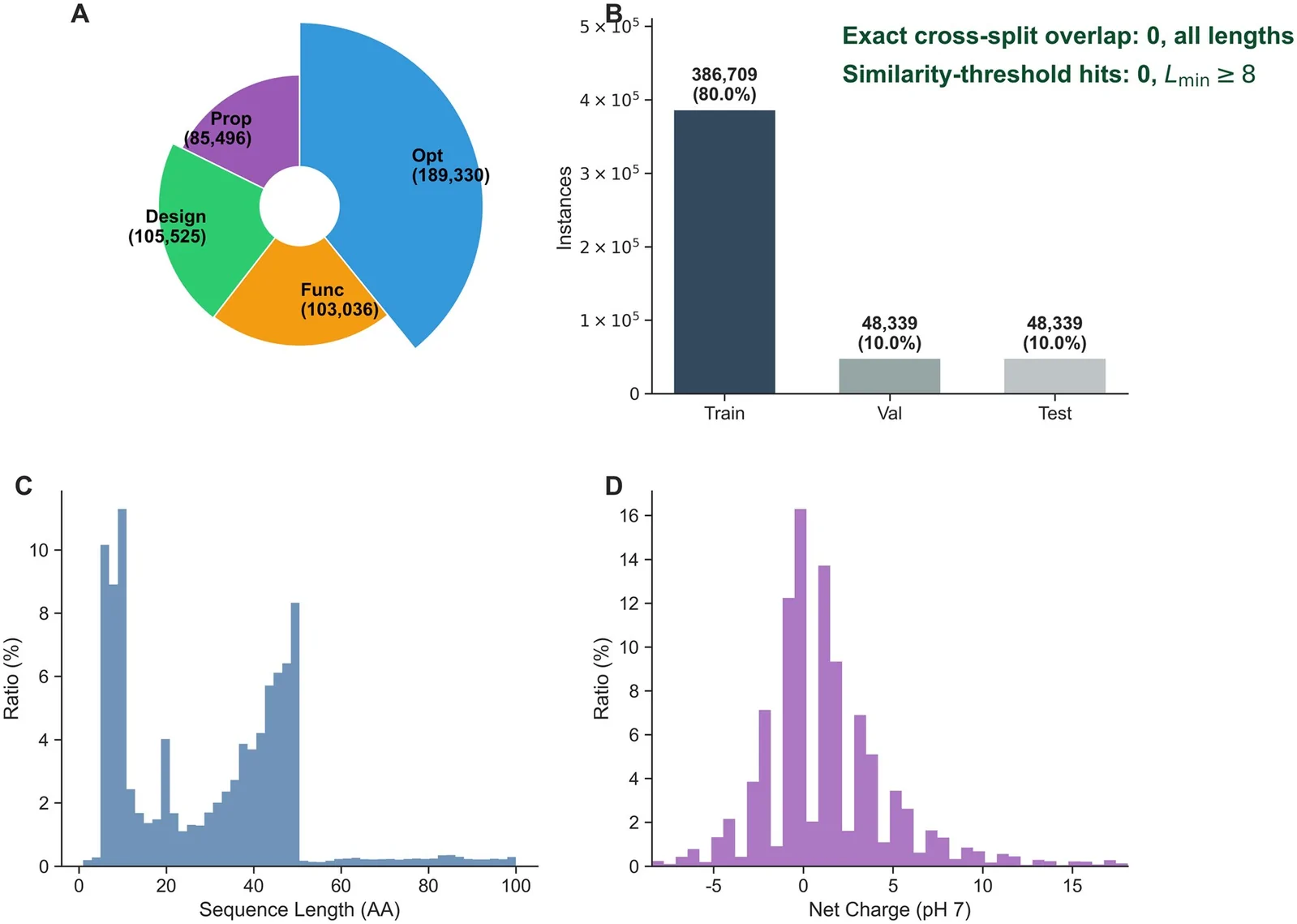
Overview of the re-partitioned Pep-Instructions dataset under the unified and leakage-controlled split protocol. (A) Task composition across the four task categories: optimization, function description, sequence design, and property prediction. (B) Split-size ratios for the training, validation, and test sets, showing an 8:1:1 partition together with no identical canonical sequences across splits for all lengths, and no cross-split pairs satisfying the predefined similarity criteria for $L_{\text{min}}\geq 8$. (C) Global sequence-length distribution of peptides in the dataset. (D) Net-charge distribution of peptides at pH 7.

Building upon this four-task formulation, we further re-partitioned the full Pep-Instructions dataset using a unified and leakage-controlled split protocol. As shown in Fig. 2, the re-partitioned Pep-Instructions dataset preserves broad coverage across all four task categories while enforcing a unified and leakage-controlled split. The overall task composition remains well represented at the corpus level (Fig. 2A), with optimization, design, function description, and property prediction all contributing substantial numbers of instances.

At the sequence level, the dataset spans broad sequence and physicochemical regimes, as reflected by the global sequence-length distribution (Fig. 2C) and net-charge distribution at pH 7 (Fig. 2D). Importantly, the new split follows a clear and consistent train/validation/test ratio of 8:1:1 (Fig. 2B), while maintaining zero cross-split exact overlap and no cross-split pairs satisfying the predefined similarity criteria for L_min≥8.

Additional dataset statistics, including endpoint- and split-specific label compositions (Table S2, available as supplementary data at *Bioinformatics* online) and sequence-length and physicochemical distributions (Figs S4 and S5, available as supplementary data at *Bioinformatics* online), are provided in the Supplementary Information. Overall, Pep-Instructions covers broad sequence-length, compositional, and physicochemical regimes. Task-wise sequence-length distributions differ substantially, with optimization and property prediction exhibiting broader and more long-tailed profiles, whereas design and function-related sequences are more concentrated in the mid-length regime. Across the full dataset, the mean isoelectric point, net charge, hydrophobicity, and instability index are 8.02, 1.32, −0.31, and 40.24, respectively. Amino-acid composition analysis shows broad coverage of all 20 canonical residues, and the overall Shannon entropy of the normalized amino-acid distribution reaches 2.9214 nats, close to the theoretical maximum of ln20≈2.996, indicating high compositional diversity. Supplementary embedding-based analyses, including UMAP visualization, further suggest that although the task subsets exhibit structured differences in representation space, the dataset as a whole retains broad diversity and coverage.

### 3.2 Function description performance

Function description prediction evaluates whether peptide sequences can be translated into standardized GO functional descriptions. As shown in Table 1, our fine-tuned model consistently outperforms all general-purpose baselines across both lexical and semantic evaluation metrics, indicating substantially improved functional description generation after domain-specific instruction tuning.

**Table 1:** Performance comparison on the function description task.^a^

	**Lexical metrics**			**Semantic metrics (BERTScore)**		
**Model**	**BLEU**	**ROUGE-L**	**Exact Match**	**Precision**	**Recall**	**F1**
GPT-3.5	0.0051	0.0905	0.0138	0.7834	0.8201	0.8012
Galactica-6.7B	0.0040	0.1280	0.0015	0.8073	0.8102	0.8086
LLaMA2-7B	0.0131	0.1325	0.0052	0.8057	0.8180	0.8117
**Ours**	**0.3348**	**0.5399**	**0.0635**	**0.8999**	**0.8860**	**0.8926**

a Precision, recall, and F1 are calculated using BERTScore to evaluate semantic alignment with the reference biological descriptions. Bold values indicate the best performance. GPT-3.5-Turbo values are from the primary recorded run; three-call repeatability and bootstrap confidence intervals are reported in Table S3, available as supplementary data at *Bioinformatics* online. Locally evaluated greedy models produced identical results across three runs.

Table 1 reports two complementary groups of metrics. The first group measures lexical fidelity, including *ROUGE-L*, *BLEU*, and *Exact Match*. Under these criteria, the baseline models achieve uniformly low scores, suggesting limited ability to reproduce standardized GO-style descriptions from peptide sequences. In contrast, the fine-tuned model yields clear gains across all lexical metrics and surpasses the strongest baseline by a substantial margin. Although the absolute values remain constrained by the difficulty of exact GO term reconstruction, the relative improvement indicates a markedly stronger capacity to generate terminology aligned with reference annotations.

The second group measures semantic alignment, using *BERTScore* Precision, Recall, and F1-Score. On these metrics, the baseline models retain partial semantic relevance but remain less consistent in capturing the specific functional content required for accurate annotation. Our model achieves the best overall performance across all three semantic indicators, showing that the generated descriptions remain better aligned with the reference functions even when wording is not strictly identical. Taken together, these results show that instruction tuning improves both the textual fidelity and the semantic faithfulness of peptide function description generation. Peptide-level bootstrap analysis further supported these improvements: paired 95% confidence intervals excluded zero for all six lexical and semantic metrics, including improvements of 0.4074 (95% CI: 0.4038–0.4112) in ROUGE-L and 0.0809 (95% CI: 0.0802–0.0816) in BERTScore-F1 over the strongest corresponding baselines (Table S3, available as supplementary data at *Bioinformatics* online).

### 3.3 Peptide design performance

**Figure 3:**
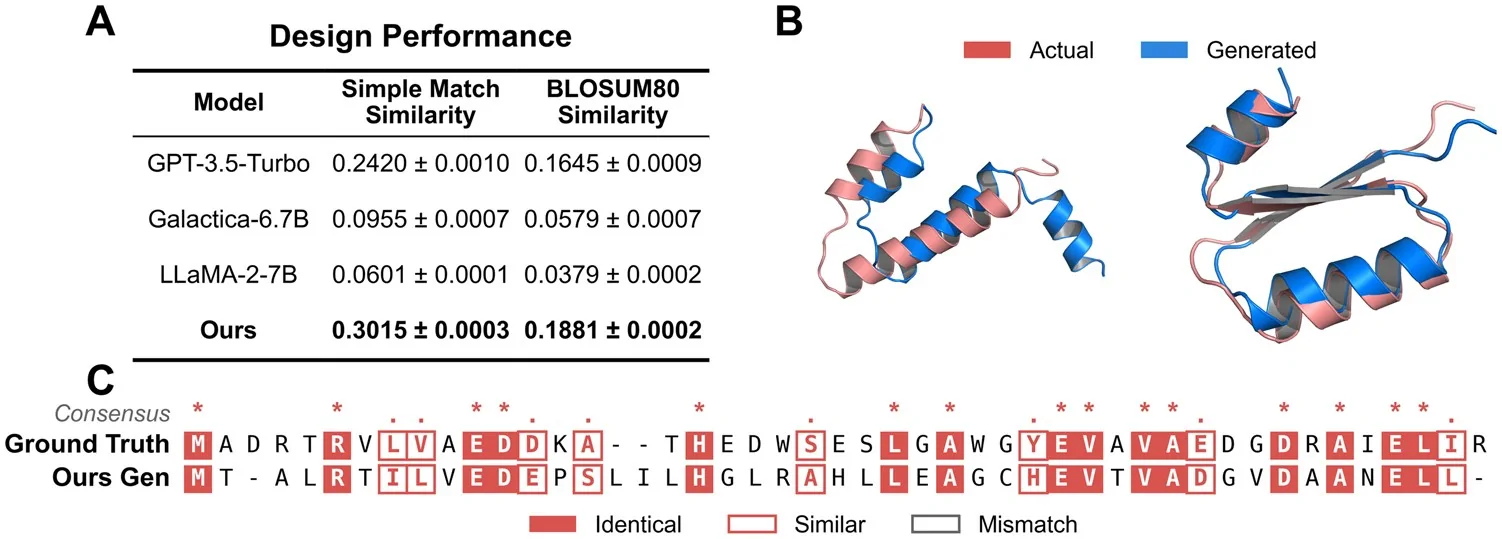
Evaluation of design performance. (A) Comparison of Simple Match and BLOSUM80 similarity scores, reported as mean ± standard deviation across three independent decoding seeds. (B) 3D superpositions of generated (blue) and ground truth (red) peptide backbones for two representative cases. (C) Sequence alignment corresponding to the second structure (right) in (B), highlighting identical residues and biochemical consensus. High BLOSUM80 scores and consensus markers indicate structural conservation.

The peptide design task assesses whether the model can generate amino acid sequences that satisfy prescribed functional and structural constraints. As shown in Fig. 3A, the fine-tuned model outperforms all baseline large language models on both sequence-identity and *BLOSUM80*-based similarity, indicating improved performance in constrained peptide generation.

Using simple match as the primary sequence-level metric, the generated peptides from the fine-tuned model show higher similarity to the reference sequences than those produced by the baseline models. Given that peptide design is a one-to-many generation problem, exact residue-level agreement is not required for a valid design. Even so, the higher sequence identity indicates that the fine-tuned model more often produces peptides that are better aligned with the reference sequences under the prescribed design constraints.

We next evaluated similarity using length-normalized *BLOSUM80*, which captures conservative substitutions beyond exact residue matching. The fine-tuned model again achieves the best performance (Fig. 3A), showing that its outputs more often preserve biochemically compatible residue patterns. This result suggests that the improvement is not limited to superficial sequence overlap.

To examine whether sequence-level similarity is accompanied by structural consistency, we predicted the structures of generated peptides using ESMFold (Lin *et al.* 2023) and superimposed them with the corresponding reference structures. Representative examples are shown in Fig. 3B and C. In these cases, the generated peptides retain similar backbone conformations and show multiple identical or conservative substitutions in the aligned sequences. Together, these results support that instruction tuning improves peptide design quality at both the sequence and structure levels.

To assess constraint-count sensitivity without retraining, we evaluated the same 149 held-out peptides using nested instructions with k=2, 4, and 6 attributes across three random seeds, while keeping all other settings unchanged. From k=2 to k=6, the valid-peptide rate decreased from 96.20% to 78.97%, and the absolute length deviation increased from 6.72 to 11.67 residues. This pattern is consistent with a greater simultaneous-constraint burden, potentially compounded by the scarcity of high-count annotations; therefore, the analysis is treated as an inference-time stress test rather than evidence that six is optimal (Table S4, available as supplementary data at *Bioinformatics* online).

### 3.4 Property prediction performance

**Figure 4:**
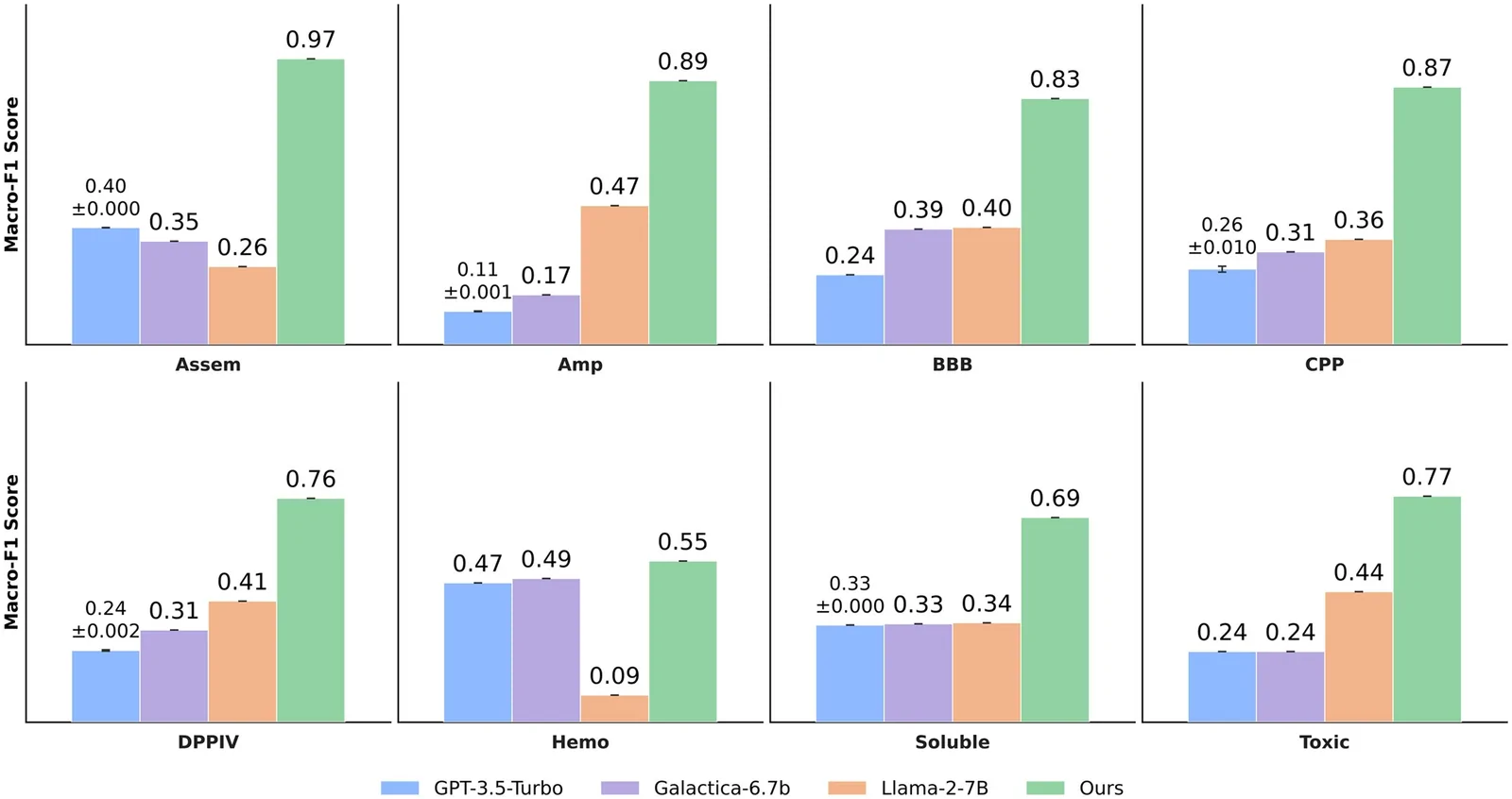
Comparison of model performance on the *property prediction* task. The figure illustrates the Macro-F1 scores of different models across eight peptide property types: *Assem*, *Amp*, *BBB*, *CPP*, *DPPIV*, *Hemo*, *Soluble*, and *Toxic*. GPT-3.5-Turbo values are mean ± SD over three API calls.

The peptide property prediction task evaluates whether a peptide sequence exhibits a specified functional property. As shown in Fig. 4, the fine-tuned model achieves the best Macro-F1 score across all eight evaluated properties. The corresponding accuracy results are provided in Table S5, available as supplementary data at *Bioinformatics* online.

The strongest absolute performance is observed for *Assem*, followed by *Amp* and *CPP*. Performance is lower for *Hemo* and *Soluble*, indicating greater difficulty for these endpoints. Averaged equally across the eight endpoints, our model achieved a *Macro-F1* of 0.7907 (95% CI: 0.7654–0.8149), compared with 0.3477 for the strongest baseline. The paired improvement was 0.4429 (95% CI: 0.4173–0.4696) (Table S3, available as supplementary data at *Bioinformatics* online).

This performance pattern is also reflected in the embedding-space visualization shown in Fig. S6, available as supplementary data at *Bioinformatics* online. Properties such as *Amp*, *CPP*, and *Toxic* show clearer positive–negative separation, whereas *Soluble*, and *Hemo* display greater class overlap. Overall, these results indicate that instruction tuning improves peptide property prediction across multiple peptide property endpoints, although the magnitude of improvement varies by task. In addition, the relatively small size of the BBB training set may also be associated with the comparatively lower performance observed for this endpoint.

### 3.5 Optimization performance on physicochemical properties

This task evaluates whether a model can edit an input peptide toward a specified physicochemical direction. As shown in Table 2, our model substantially improves directional success rates over general-purpose LLM baselines under all similarity thresholds. At the 30% and 50% similarity thresholds, it achieves near-complete success across all seven tasks, and it remains the strongest LLM-based method at the more stringent 70% threshold.

**Table 2:** Directional success rates (%) for physicochemical optimization against general-purpose LLM baselines.^a^

Task type	**Galactica-6.7B**			**GPT-3.5-Turbo**			**LLaMA-2-7B**			**Ours**		
	30%	50%	70%	30%	50%	70%	30%	50%	70%	30%	50%	70%
Instability index ↓	40.5	37.6	32.6	30.4	30.4	30.1	30.1	29.6	24.9	**99.6**	**98.0**	**35.1**
Isoelectric point ↑	26.2	25.8	23.0	36.2	36.2	35.7	29.9	24.7	17.1	**99.7**	**99.5**	**55.5**
Isoelectric point ↓	20.2	17.8	14.0	37.3	37.3	37.1	15.6	13.4	9.6	**100.0**	**99.9**	**62.0**
Charge ↑	32.5	31.5	27.5	52.7	52.6	52.2	35.1	33.5	27.8	**99.8**	**99.6**	**44.2**
Charge ↓	18.6	17.0	13.6	27.5	27.5	27.4	17.7	15.1	11.7	**100.0**	**99.8**	**40.8**
Log *P* ↑	44.5	43.3	38.9	39.4	39.4	39.1	7.6	7.4	7.0	**100.0**	**99.7**	**53.8**
Log *P* ↓	36.4	34.1	27.7	46.4	46.4	46.0	17.0	16.3	14.5	**99.9**	**99.6**	**58.8**

a Success rate denotes the percentage of samples achieving the target modification direction (↑ = increase; ↓ = decrease) under each similarity threshold. This table reports comparisons among LLM-based methods only. Bold indicates the best LLM performance.

These gains cover instability index, isoelectric point, net charge, and Log*P*, indicating that peptide-specific instruction tuning markedly improves property-oriented editing relative to untuned or general-purpose LLMs. Notably, directional success rate captures only whether the target property changes in the desired direction, and therefore does not fully reflect the magnitude or consistency of optimization.

To provide a more discriminative evaluation, we additionally report successful-sample effect sizes ($\delta^{+}$) and full-sample signed net shifts (Δ along the target direction) in Table 3. Under these stricter criteria, our model achieves substantially larger effect sizes and overall directional improvements than all general-purpose LLM baselines (median $\delta^{+}$: 2.998; mean $\delta^{+}$: 15.300; median Δ: 2.998; mean Δ: 15.287), indicating stronger and more consistent property modulation. Representative examples are shown in Fig. S7, available as supplementary data at *Bioinformatics* online. In an additional full-sample analysis without imposing an extra sequence-similarity cutoff, source-peptide bootstrap resampling showed that the improvements over the strongest baseline ranged from 47.3 to 72.5 percentage points across the seven property–direction settings (Table S3, available as supplementary data at *Bioinformatics* online).

**Table 3:** Effect-size and full-sample net-shift statistics for physicochemical optimization.^a^

Model	Succ. (%)	**Median** $\delta^{+}$	**Mean** $\delta^{+}$	**Median** Δ	**Mean** Δ
GPT-3.5-Turbo	41.38	0.460	3.771	0.000	0.1981
Galactica-6.7B	36.3	0.404	6.289	0.000	0.198
LLaMA-2-7B	37.4	1.000	6.694	0.000	0.752
Ours	**99.9**	**2.998**	**15.300**	**2.998**	**15.287**

a $\delta^{+}$ denotes the effect size among successful samples, while Δ represents the signed full-sample displacement along the target direction. These metrics provide a more discriminative assessment of optimization quality beyond binary success rate. The bold values indicate the best performance for each metric.

### 3.6 Exploratory evaluation of functionalproperty editing

Building on the model’s performance in physicochemical optimization, we conducted a more challenging exploratory evaluation on functional-property editing. We refer to the direct generation setting as zero-shot-style because the model was fine-tuned on sequence–label supervision without paired sequence-to-sequence editing examples or explicit optimization trajectories. To assess this setting in a scalable and internally consistent manner, generated candidates were evaluated using an independently trained property-prediction oracle (Table S6, available as supplementary data at *Bioinformatics* online), rather than wet-lab validation.

**Figure 5:**
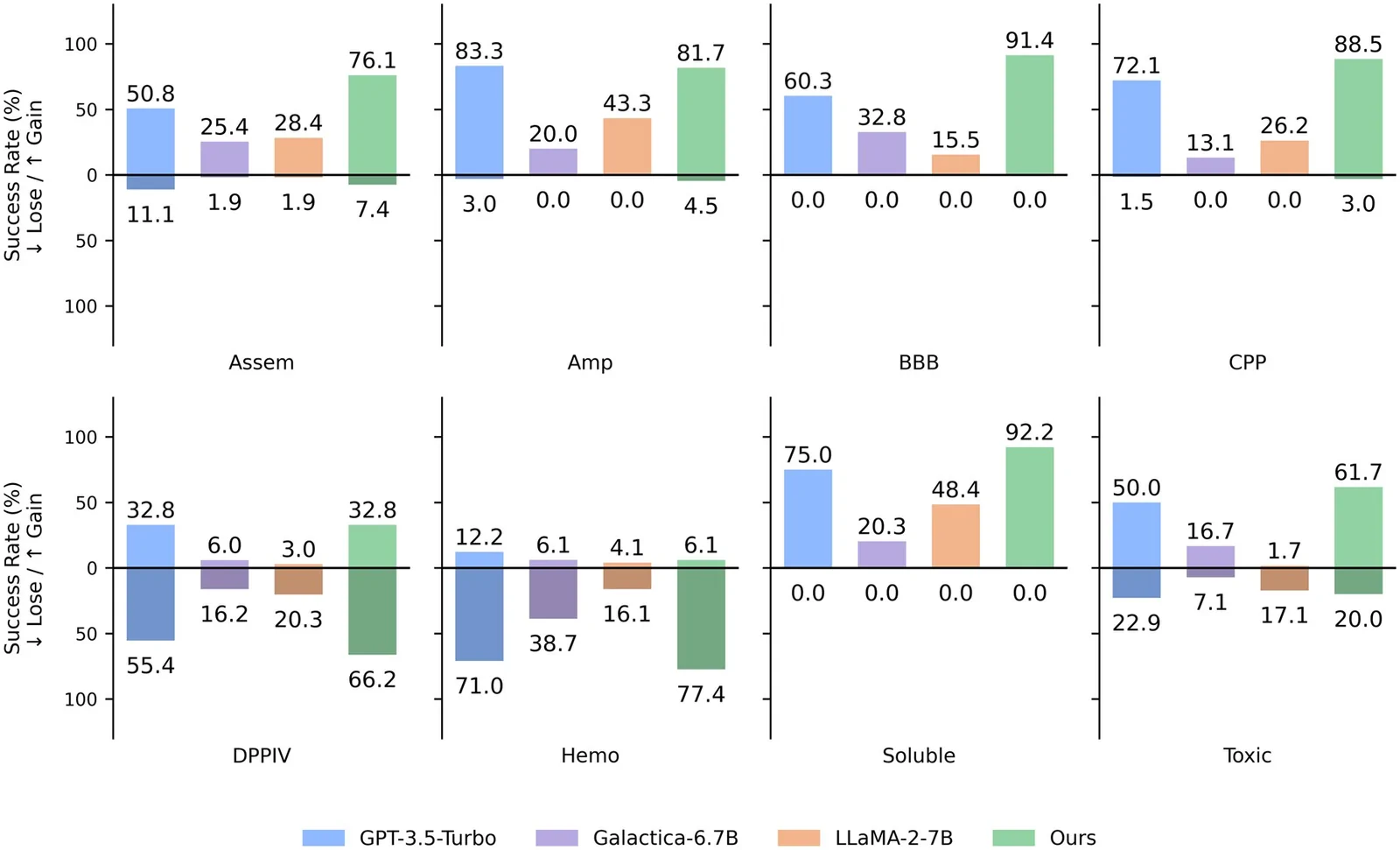
Success rates of exploratory functional editing at a 50% sequence-similarity threshold. “Gain” and “Lose” denote oracle-predicted label changes from negative to positive and from positive to negative, respectively.

To characterize model behavior more systematically, we divided the evaluation into two trajectories: predicted gain, which changes a property-negative input into a surrogate-predicted positive candidate, and predicted loss, which changes a property-positive input into a surrogate-predicted negative candidate. Comprehensive success rates under different similarity thresholds are reported in Table S7, available as supplementary data at *Bioinformatics* online, and representative results at the 50% similarity threshold are shown in Fig. 5. Under this oracle-based evaluation protocol, the instruction-tuned 7B model outperformed GPT-3.5-Turbo in most evaluated property endpoints, suggesting that domain-specific instruction tuning may provide an advantage for peptide-oriented functional editing.

We also observed a marked asymmetry between the predicted-gain and predicted-loss editing trajectories. Predicted gain appears easier for properties associated with relatively local sequence ingredients, whereas more globally organized functions remain harder to induce under strict similarity constraints. Conversely, predicted loss is often more successful for fragile functional patterns, where limited perturbations can disrupt key sequence determinants. These observations are consistent with the hypothesis that some functional properties are locally inducible, whereas others depend on more distributed sequence organization.

Finally, we examined whether conditional refinement could improve outputs when direct generation was insufficient. Specifically, candidates that failed to satisfy the oracle criterion after the initial generation were subjected to targeted reprompting, with up to two additional guided attempts. We therefore distinguish this refined setting from strict one-pass zero-shot generation and interpret it instead as test-time conditional refinement. As summarized in Table 4, this multi-step procedure improved oracle-based editing performance across all evaluated properties. Overall, these results provide preliminary *in silico* evidence that instruction-tuned generation can generate sequences satisfying the oracle-defined editing criterion, while also highlighting the need for stronger validation, oracle-isolation analysis, and external or experimental confirmation in future work.

**Table 4:** Oracle-estimated performance of direct and conditionally refined functional editing across peptide properties.^a^

Method	Assem	Amp	BBB	CPP	DPPIV	Hemo	Soluble	Toxic
Direct	0.554	0.524	0.526	0.523	0.539	0.523	0.488	0.515
Refined^b^	**0.603**	**0.548**	**0.561**	**0.570**	**0.674**	**0.613**	**0.496**	**0.662**

a Values denote target-label attainment rates estimated by the learned evaluation oracle for exploratory functional editing. Higher values indicate better alignment with the target editing objective under the adopted *in silico* evaluation protocol. The bold values indicate the best performance for each metric.

b Refined generation follows a conditional three-step procedure: the model first produces a direct sequence; if it fails to satisfy the target property, a second sequence is generated using one reference for guidance; upon a second failure, a third sequence is generated using an alternative reference. This setting is therefore interpreted as test-time conditional refinement rather than strict one-shot zero-shot-style generation.

## 4 Discussion

In the task of peptide function description, our framework demonstrates clear superiority over baselines, achieving substantial gains in both lexical (e.g. ROUGE) and semantic metrics (e.g. BERTScore). This performance pattern reveals a specific learning outcome: the high lexical scores reflect the model’s robust adherence to the Structured Scientific Instructions established in our framework. Crucially, while the lower keyword-based Exact Match rate implies that specific terminological selection may diverge from ground truth, the consistently high BERTScore confirms that the model preserves the core functional logic. This suggests that instruction-tuning empowers the model to generate structurally professional and semantically coherent descriptions, effectively decoding dense peptide bio-information even without perfect terminological memorization.

The interpretation of these results necessitates contextualization within the high-dimensional complexity of the Gene Ontology landscape and the “Open World” annotation challenge, where the vast, hierarchical label space causes strict lexical metrics to underestimate utility by penalizing unverified valid descriptions. To further characterize upper-tail behavior, we conducted a descriptive model-wise top-*K* analysis (Fig. S8, available as supplementary data at *Bioinformatics* online), ranking test samples independently for each model by its per-sample F1 score and recomputing average F1 on the corresponding top 3000, 1000, 500, and 100 subsets. Under this post hoc best-case view, baseline models plateau quickly, whereas the instruction-tuned model exhibits a steeper rise and a higher apparent upper bound. As the ranked subsets are model-specific rather than shared, this analysis is descriptive rather than a formal comparison; nevertheless, the observed pattern is consistent with improved capacity for high-quality peptide function description on favorable samples.

In the specific context of property prediction, comparative analysis highlights distinct response biases rooted in the training paradigms of baseline models. GPT-3.5-Turbo exhibits a conservative, negative response pattern driven by reinforcement learning from human feedback (RLHF) safety constraints, whereas LLaMA2 and Galactica (Taylor *et al.* 2022) display affirmative biases attributed to instruction compliance and declarative literature training, respectively. These observations suggest that without specific domain alignment, general-purpose LLMs rely heavily on their pretrained inductive biases—ranging from safety-induced skepticism to hallucinated existence statements—rather than grounding their outputs in biochemical reality.

Crucially, our results uncover a structural gap: enhanced discriminative representation does not automatically translate into effective generative optimization. We attribute this limitation to four factors: the divergence between static predictive mapping and directional evolutionary objectives; gradient interference where discriminative stability conflicts with generative flexibility; the absence of paired trajectories to learn modification pathways; and the tendency of instruction tuning to suppress latent space exploration required for novel solutions. Future work must bridge this gap by integrating paired optimization datasets, transitioning the model from simply “identifying the good” to “producing the better.”

## 5 Conclusion

In summary, we present a unified peptide instruction-tuning framework that adapts a general-purpose large language model to peptide understanding, design, prediction, and sequence refinement. By constructing Pep-Instructions and enforcing a unified sequence-level split with exact deduplication and length-aware similarity control for sequence pairs with $L_{\text{min}}\geq 8$, we establish a more reliable evaluation setting for peptide-centric instruction tuning. Within this framework, LoRA-based adaptation of LLaMA2-7B yields consistent improvements over general-purpose baselines across function description, sequence design, property prediction, and physicochemical optimization, using raw peptide sequences alone and without explicit structural or evolutionary priors. At the same time, the current results on functional editing remain preliminary, as they are supported by oracle-based *in silico* evaluation and test-time conditional refinement rather than experimental validation. Overall, these findings indicate that peptide-specific instruction tuning provides a promising computational framework for transferring general linguistic competence into peptide modeling, while future work should focus on stronger generative optimization supervision, reduced oracle dependence, and external or experimental validation of functional editing in biologically relevant settings.

## Supplementary Material

btag618_Supplementary_Data

## Data Availability

The source code and the curated Pep-Instructions dataset generated during this study are publicly available at https://github.com/kjY7836/pepinstruction. The fine-tuned model weights of Pep-Instructions are available at https://huggingface.co/Codelife176/Pep-instruction. All resources are open-access, and instructions for deployment and evaluation can be found on our GitHub homepage.
