# Supplementary material for: Bridging linguistic reasoning and biophysical reality toward peptide engineering via instruction-tuned language modeling
Source: Bioinformatics. 2026 Aug 17;42(9):btag618. doi: 10.1093/bioinformatics/btag618 (PMC13529362; doi:10.1093/bioinformatics/btag618)
Supplement: Supplementary file 1 [file btag618_supplementary_data.pdf]

Supporting Information for: **Bridging Linguistic Reasoning and Biophysical Reality Toward Peptide Engineering via Instruction-Tuned Language Modeling**

Kaijun Yang[1], Tianxiang Wu[1], Wenbo Zhang[1*], Pengyong Li[1*]
School of Computer Science and Technology, Xidian University, Xi'an 710126, Shaanxi, China

Correspondence to: zhangwenbo01@xidian.edu.cn, lipengyong@xidian.edu.cn

Feature	Template (Design Description)
Function [CC]	For general function, the protein needs to meet that {function}.
Catalytic activity	The protein should be designed to catalyze the following reaction {catalytic activity}.
Gene Ontology (biological process)	The designed protein must be able to regulate {biological process}.
Gene Ontology (cellular component)	The designed protein localizes to the {cellular component}.
Gene Ontology (molecular function)	The protein must be able to {molecular function}.
Cofactor	The protein must bind to {cofactor} in order to perform its enzymatic function.
Pathway	A protein that is capable of catalyzing reactions within the {pathway} with high efficiency and specificity.
Domain [FT]	The target protein must incorporate the {domain} domain.
Motif	The protein's functional design necessitates the inclusion of a {motif}.
Signal peptide	Incorporate a signal peptide in the protein design.
DNA binding	The designed protein must have DNA-binding capability.
Transmembrane	The protein must contain one or more transmembrane regions.

Figure S1. Schematic of the template-mapping process for design task. Functional and structural attributes—such as GO annotation, Domain, Catalytic activity, and Pathway—are extracted from the UniProt database and transformed into task-specific instruction templates to guide the peptide design generation.

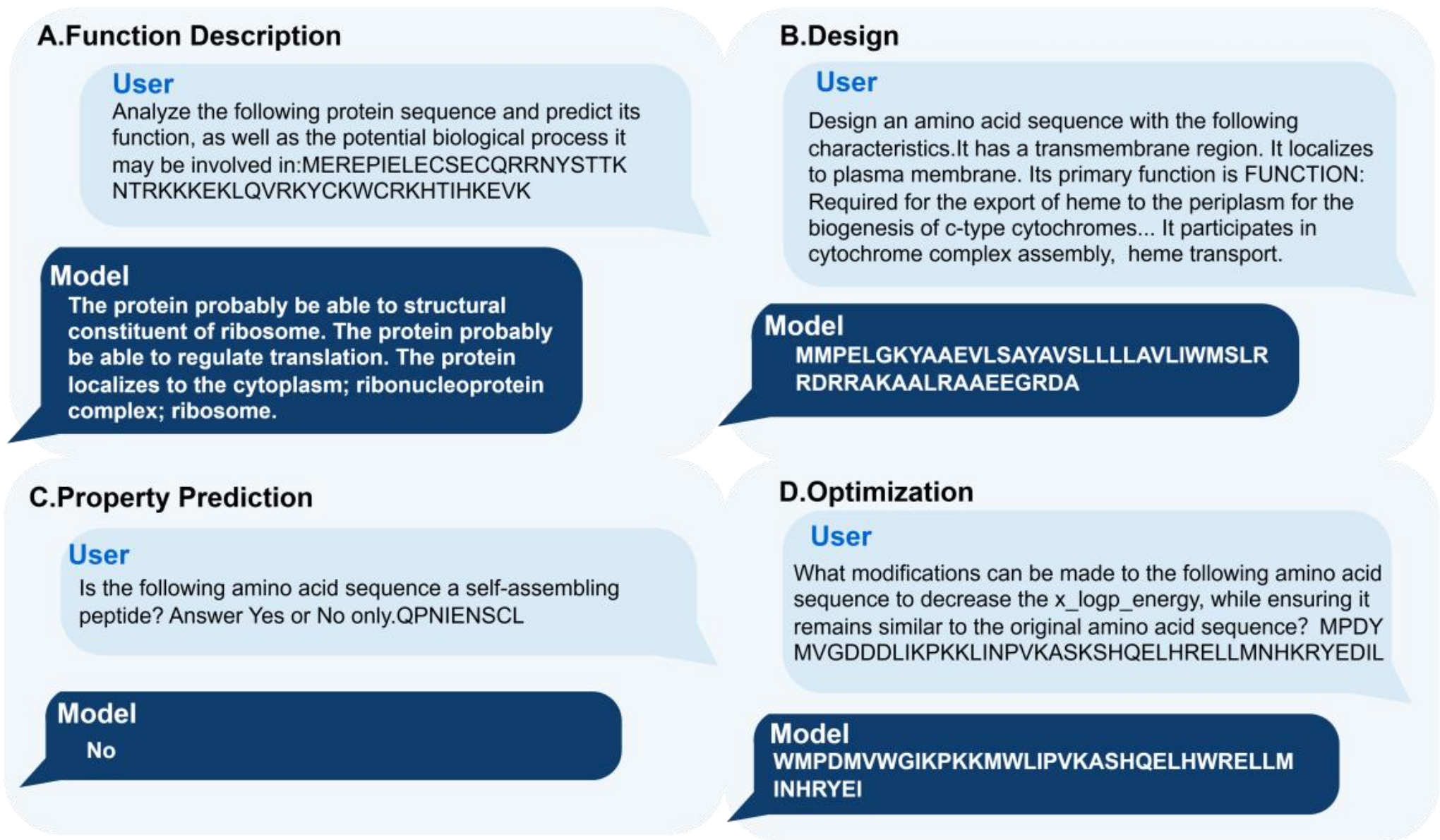

Figure S2. Representative instruction-response pairs for the four peptide tasks. (A) Function Description: Infers biological roles from amino acid sequences. (B) Sequence Design: Generates peptides based on specific functional or structural constraints. (C) Property Prediction: Classifies specific peptide traits (e.g., self-assembly) via binary outputs. (D) Optimization: Modifies sequences to enhance physicochemical properties while preserving sequence similarity.

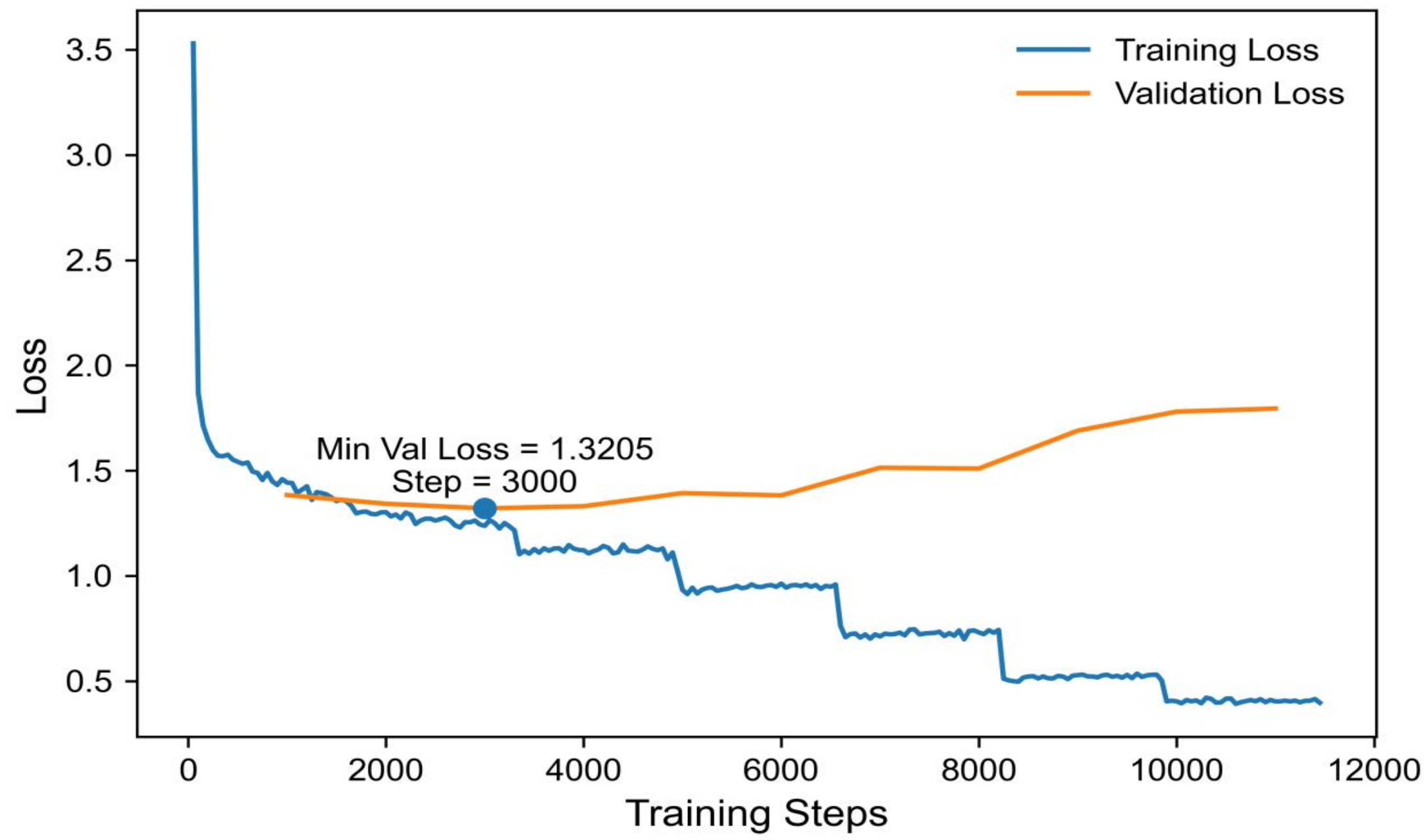

Figure S3. Training convergence and model selection. The learning curves show the Cross-Entropy Loss on training (blue) and validation (orange) sets. The model demonstrates stable convergence, with the optimal checkpoint selected at step 3,000 (Min Val Loss = 1.3205) to maximize generalization and prevent overfitting.

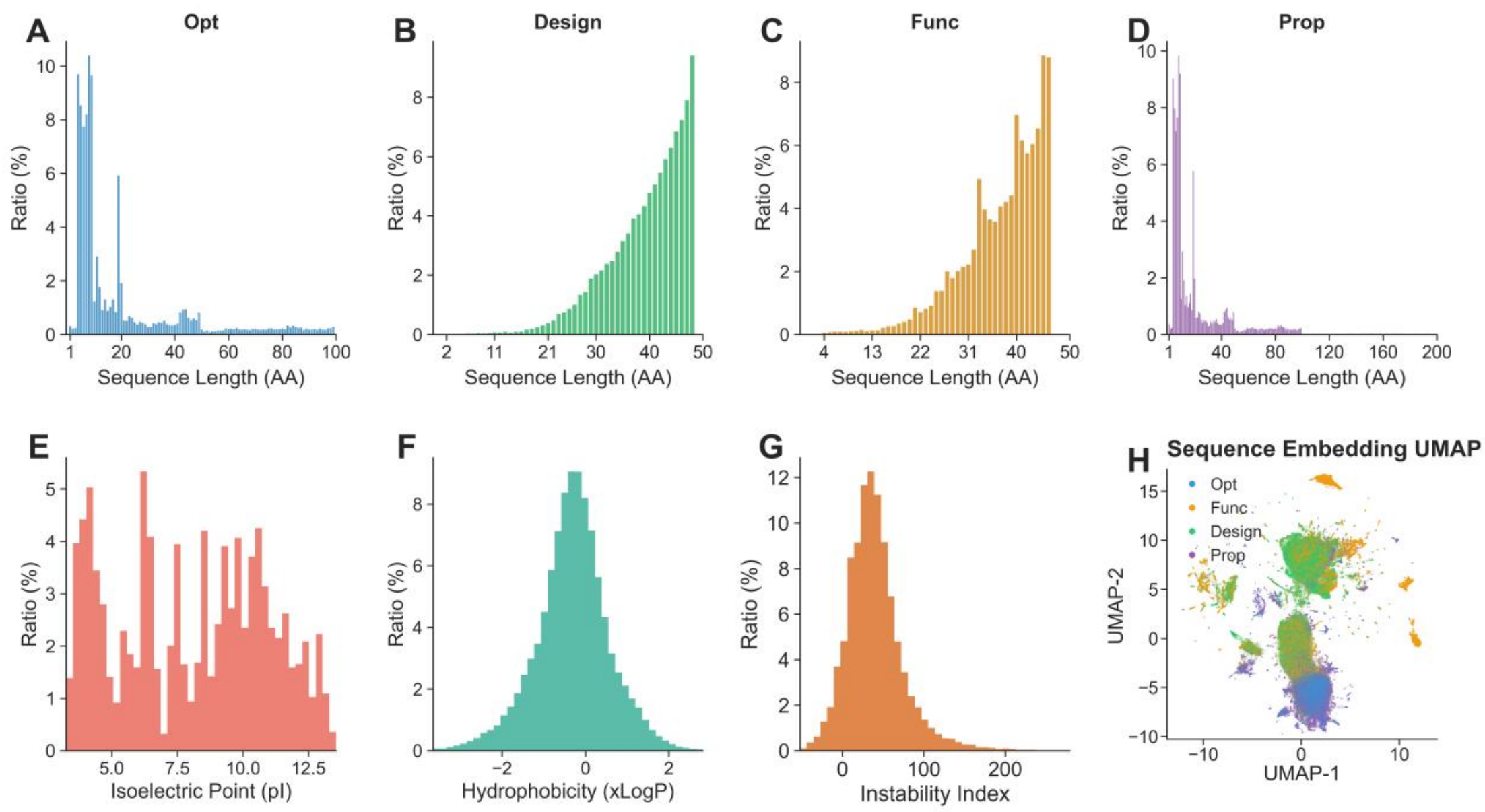

Figure S4. Statistical characterization of the Pep-Instructions dataset. (A–D) Task-wise sequence-length distributions for optimization, sequence design, function description, and property prediction. (E–G) Distributions of key physicochemical properties (pI, hydrophobicity, instability index), indicating broad biochemical coverage. (H) UMAP projection of sequence embeddings, showing wide latent-space coverage with partial task-wise clustering. Pairwise KL divergence further quantifies inter-task differences: Design–Function (17.13) and Function–Property (16.12) show substantial divergence, whereas Design–Property (1.37) is markedly closer.

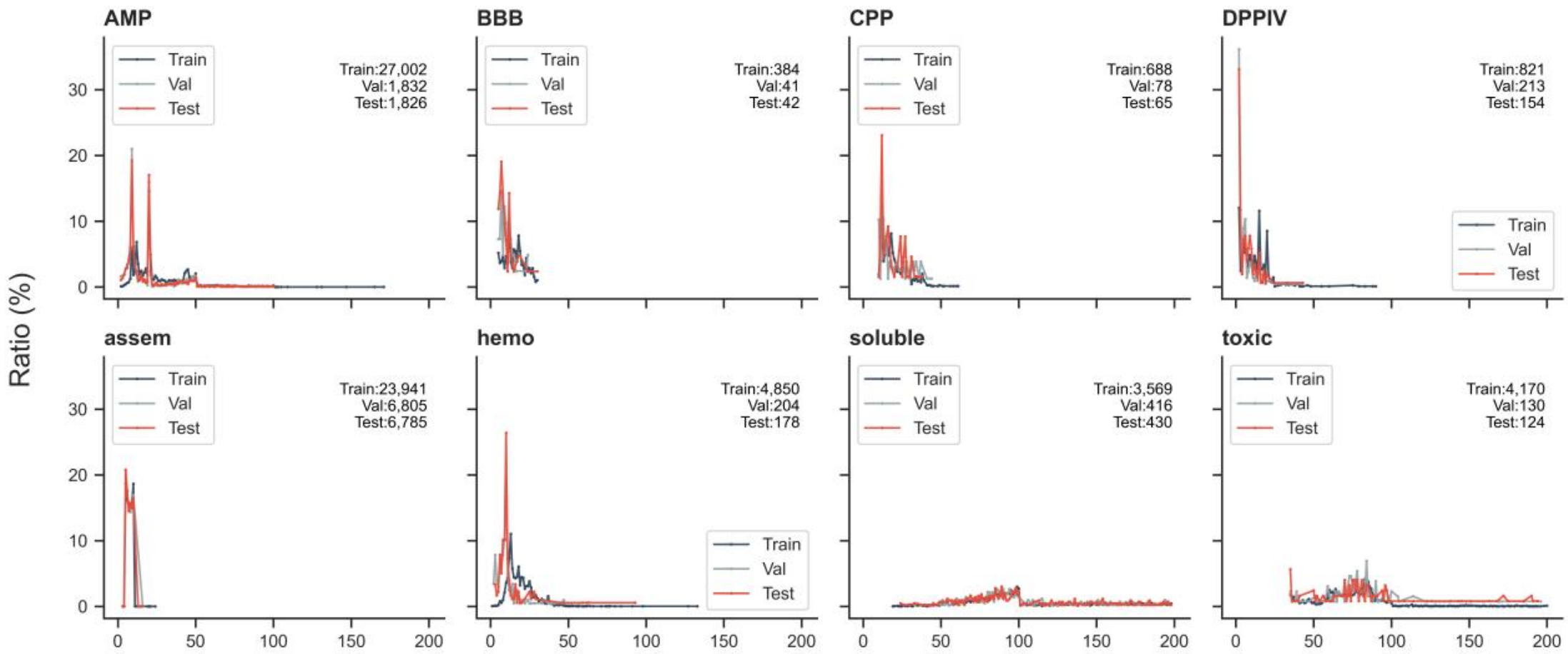

Figure S5. Sequence length distribution of datasets for property prediction tasks. The charts illustrate the distribution of amino acid sequence lengths across eight peptide properties: Amp, BBB, CPP, DPPIV, Self-Assembly (assem), Hemolytic (hemo), Solubility (soluble), and Toxicity (toxic). The x-axis represents the sequence length, and the y-axis indicates the relative proportion (Ratio %) within each dataset. The sample size is annotated in the top-right corner of each panel.

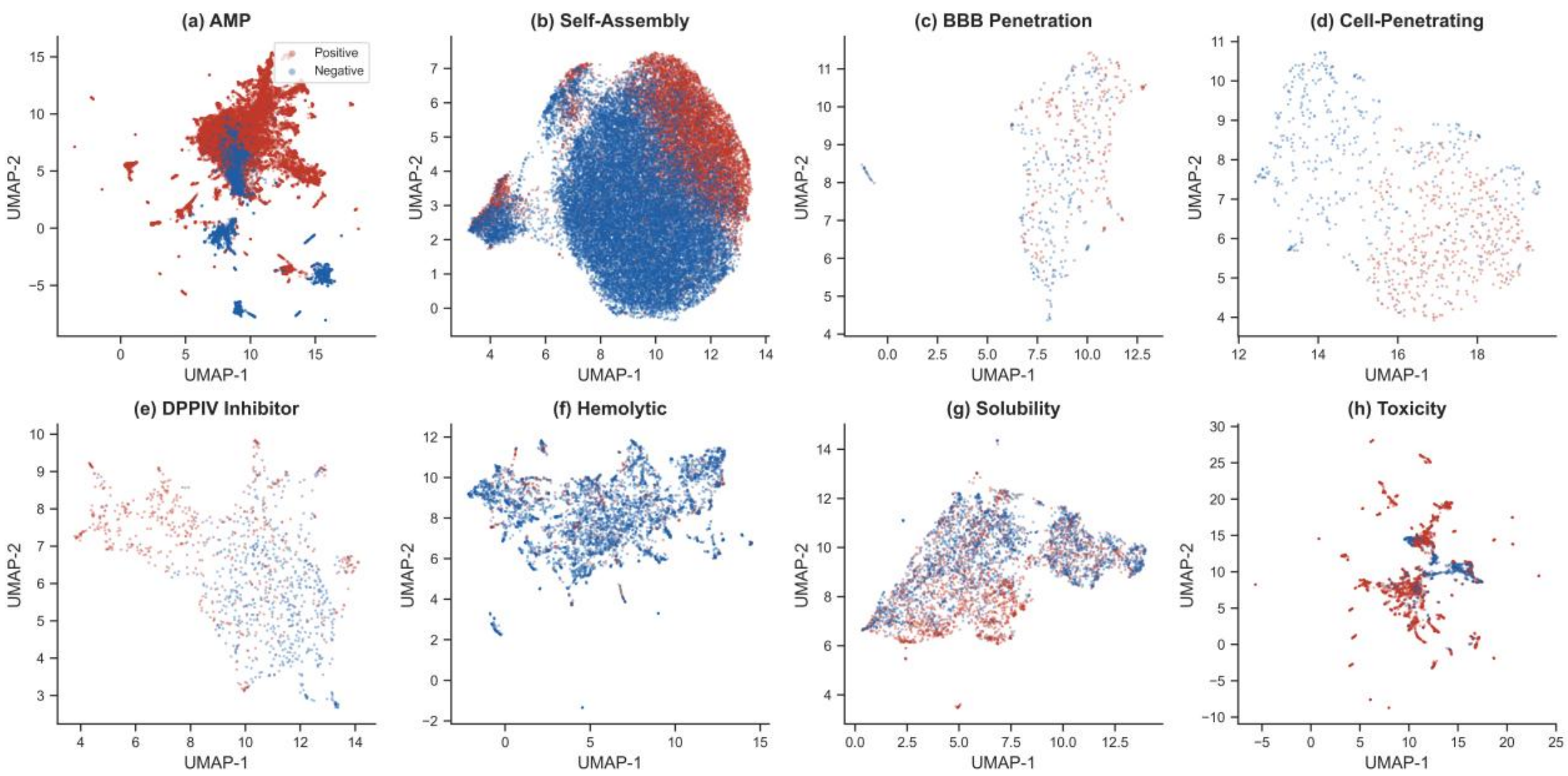

Figure S6. UMAP visualization of peptide embeddings for property prediction tasks. The 2D projections illustrate the feature space distribution for eight distinct peptide properties: (a) Amp, (b) Self-Assembly, (c) BBB Penetration, (d) Cell-Penetrating, (e) DPPIV Inhibitor, (f) Hemolytic, (g) Solubility, and (h) Toxicity. Red points represent positive samples (possessing the property), while blue points represent negative samples.

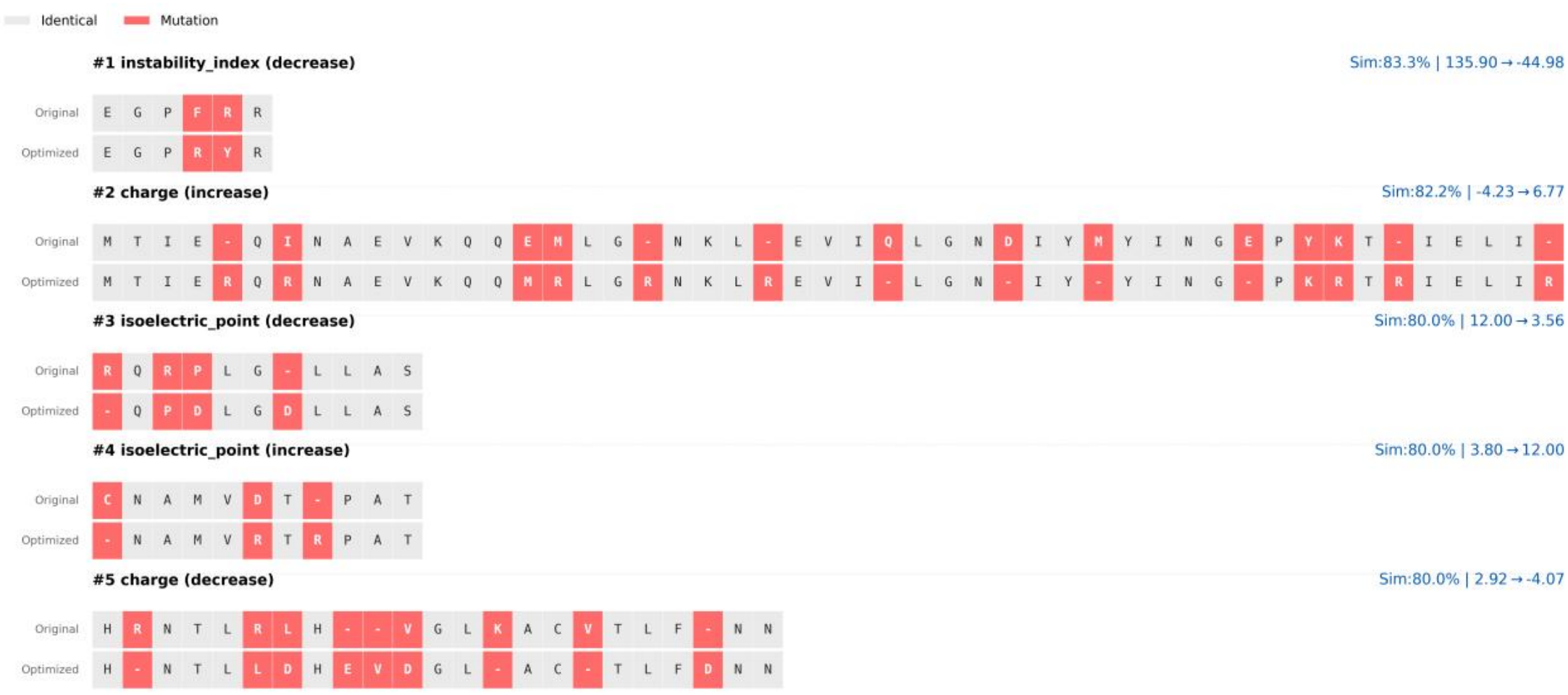

Figure S7. Optimization of peptide physicochemical properties with high sequence conservation. Top 5 representative cases for distinct property optimization tasks are shown. Gray blocks indicate identical residues, while red blocks highlight mutations introduced by the model.

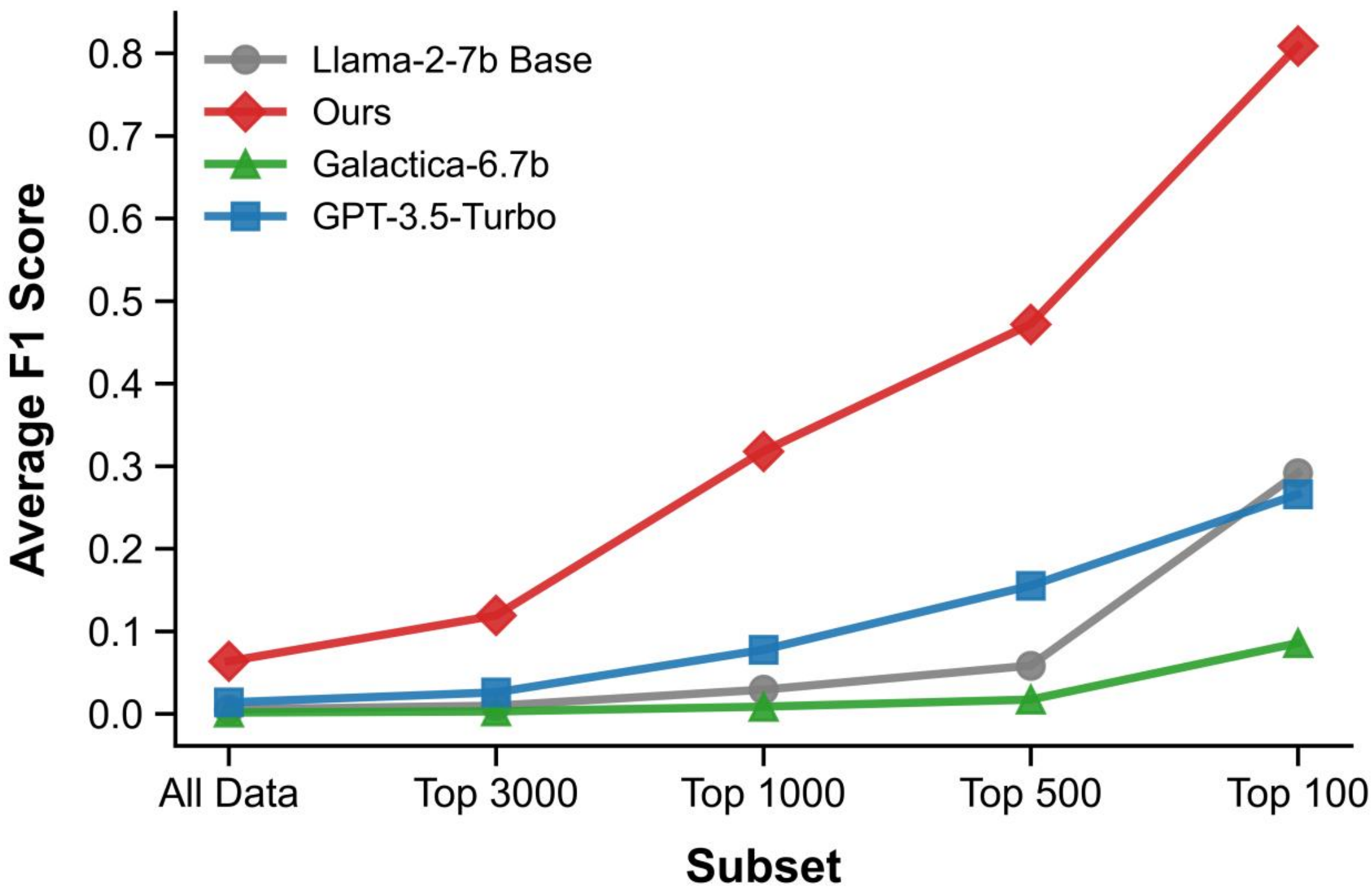

Figure S8. Stratified comparison of Exact Match F1 scores across different data subsets. The line chart displays the average F1 scores for the proposed model (Ours) compared to three baseline models. The evaluation is performed on the full dataset and progressively smaller subsets of top-ranked samples (Top 3000 to Top 100), highlighting the performance trends across different tiers of prediction quality.

Table S1. Distribution of attribute counts in the final design-test instructions (N=6,728).

Attribute count	Final instructions	Final (%)
1	242	3.6
2	2,197	32.65
3	2,371	35.24
4	1,410	20.96
5	359	5.34
6	149	2.21
**Total**	**6,728**	**100**

Table S2. Endpoint and split-specific label composition of the property-prediction benchmark before and after training-only oversampling. Pos and Neg denote the numbers of positive and negative samples before oversampling. The original property-prediction benchmark is partitioned into training, validation and test subsets. Minority-class oversampling was applied exclusively to the training subset to expand its effective volume, while validation and test sets retained their original distributions. Because homology-connected components were treated as indivisible split units, exact endpoint-wise label stratification was not enforced.

Endpoint	DPPIV	Amp	BBB	CPP	Hemo	Assem	Soluble	Toxic
Train Pos	345	22580	184	327	755	8543	1838	3750
Train Neg	476	4422	200	361	4095	15398	1731	420
Train Pos %	42.02%	83.62%	47.92%	47.53%	15.57%	35.68%	51.50%	89.93%
Val Pos	141	1595	24	52	28	2525	217	83
Val Neg	72	237	17	26	176	4280	199	47
Val Pos %	66.20%	87.06%	58.54%	66.67%	13.73%	37.11%	52.16%	63.85%
Test Pos	107	1624	29	36	18	2437	218	85
Test Neg	47	202	13	29	160	4348	212	39
Test Pos %	69.48%	88.94%	69.05%	55.38%	10.11%	35.92%	50.70%	68.55%
Oversampled Train Pos	517	22580	184	327	3020	17086	1838	3750
Oversampled Train Neg	476	22110	200	361	4095	15398	1731	3780
Oversampled Pos %	52.06%	50.53%	47.92%	47.53%	42.45%	52.60%	51.50%	49.80%

Table S3. Bootstrap confidence intervals and evaluation-time repeatability across the non-design tasks for GPT. Panels A–C report 95% percentile bootstrap confidence intervals based on 2,000 resamples and are conditional on the fixed outputs from the primary recorded evaluation run. Panels D–F report mean ± standard deviation across three independent GPT-3.5-Turbo API calls at temperature 0. Thus, Panels A–C quantify finite-test-set sampling uncertainty, whereas Panels D–F quantify variability across repeated API calls.

Panel A. Property prediction

Endpoint	Ours	Ours 95% CI	Strongest baseline	Baseline	Difference	Paired 95% CI
Assem	0.9676	[0.9632, 0.9719]	GPT-3.5	0.3953	0.5723	[0.5667, 0.5775]
Amp	0.8935	[0.8729, 0.9141]	Galactica	0.4756	0.4178	[0.3928, 0.4405]
BBB	0.8329	[0.6998, 0.9443]	Galactica	0.4085	0.4244	[0.2914, 0.5358]
CPP	0.8718	[0.7834, 0.9527]	Galactica	0.3564	0.5154	[0.4270, 0.5963]
DPPIV	0.7579	[0.6857, 0.8316]	Galactica	0.41	0.3479	[0.2758, 0.4170]
Hemo	0.5456	[0.4675, 0.6332]	GPT-3.5	0.4718	0.0737	[−0.0058, 0.1522]
Soluble	0.6907	[0.6462, 0.7347]	Galactica	0.3416	0.349	[0.3020, 0.3937]
Toxic	0.7654	[0.6944, 0.8300]	LLaMA2	0.4419	0.3235	[0.2133, 0.4321]
Overall	0.7907	[0.7654, 0.8149]	Galactica	0.3477	0.4429	[0.4173,0.4696]

Panel B. Function description

Metric	Ours	Ours 95% CI	Strongest baseline	Baseline	Difference	Paired 95% CI
BLEU	0.3348	[0.3306, 0.3390]	LLaMA2	0.0131	0.3217	[0.3177, 0.3256]
ROUGE-L	0.5399	[0.5361, 0.5438]	LLaMA2	0.1325	0.4074	[0.4038, 0.4112]
Exact Match	0.0635	[0.0595, 0.0674]	GPT-3.5	0.0138	0.0498	[0.0457, 0.0539]
BERTScore-P	0.8999	[0.8990, 0.9008]	Galactica	0.8073	0.0926	[0.0916, 0.0935]
BERTScore-R	0.886	[0.8853, 0.8868]	GPT-3.5	0.8201	0.066	[0.0653, 0.0667]
BERTScore-F1	0.8926	[0.8919, 0.8933]	LLaMA2	0.8117	0.0809	[0.0802, 0.0816]

Panel C. Success rates of Physicochemical optimization

Property-direction	Ours	Ours 95% CI	Strongest baseline	Baseline	Difference	Paired 95% CI
Instability index ↓	99.70%	[99.3%, 100.0%]	Galactica	44.00%	55.70%	[52.7%, 58.8%]
pI ↑	99.80%	[99.5%, 100.0%]	GPT-3.5	36.30%	63.50%	[60.4%, 66.5%]
pI ↓	100.00%	[100.0%, 100.0%]	GPT-3.5	37.30%	62.70%	[59.7%, 65.8%]
Charge ↑	100.00%	[100.0%, 100.0%]	GPT-3.5	52.70%	47.30%	[44.2%, 50.2%]
Charge ↓	100.00%	[100.0%, 100.0%]	GPT-3.5	27.50%	72.50%	[69.7%, 75.3%]
LogP ↑	100.00%	[100.0%, 100.0%]	Galactica	47.20%	52.80%	[49.8%, 55.9%]
LogP ↓	99.90%	[99.7%, 100.0%]	GPT-3.5	46.40%	53.50%	[50.6%, 56.4%]

Panel D. GPT-3.5-Turbo repeatability for function description.

Metric	BLEU	ROUGE-L	Exact Match	BERTScore Precision	BERTScore Recall	BERTScore F1
**Mean ± SD**	0.0052 ± 0.0001	0.0911 ± 0.0005	0.0141 ± 0.0005	0.7839 ± 0.0004	0.8207 ± 0.0005	0.8018 ± 0.0005

Panel E. GPT-3.5-Turbo repeatability for property prediction

Endpoint	Assem	Amp	BBB	CPP	DPPIV	Hemo	Soluble	Toxic
**Macro-F1, mean ± SD**	0.40 ± 0.0004	0.11 ± 0.0012	0.24 ± 0.0000	0.26 ± 0.0099	0.24 ± 0.0023	0.47 ± 0.0000	0.33 ± 0.0003	0.24 ± 0.0000

Panel F. Success rates of GPT-3.5-Turbo repeatability for physicochemical optimization

Property direction	Instability index ↓	Isoelectric point ↑	Isoelectric point ↓	Charge ↑	Charge ↓	LogP ↑	LogP ↓
**30% threshold**	30.1 ± 0.76	37.8 ± 1.42	37.7 ± 0.36	53.0 ± 0.25	26.7 ± 0.80	39.3 ± 0.12	46.3 ± 0.81
**50% threshold**	30.1 ± 0.76	37.8 ± 1.42	37.7 ± 0.36	52.8 ± 0.25	26.7 ± 0.80	39.3 ± 0.12	46.2 ± 0.86
**70% threshold**	29.7 ± 0.81	37.4 ± 1.49	37.4 ± 0.30	52.3 ± 0.15	26.6 ± 0.75	38.8 ± 0.23	45.9 ± 0.85

Table S4. Inference-time sensitivity of our model to the number of attributes in design instructions. Values are mean ± standard deviation over three matched runs on the same 149 held-out peptides, using nested k=2, 4, and 6 attribute sets. No retraining was performed, and all other settings were fixed. The reported metrics assess reference-level sequence resemblance and inference stability, not biological constraint satisfaction.

**Metric**	**(k=2)**	**(k=4)**	**(k=6)**
Validity (%)	96.20 ± 1.03	90.60 ± 1.35	78.97 ± 0.78
Identity	0.309 ± 0.003	0.301 ± 0.002	0.266 ± 0.002
BLOSUM80	0.177 ± 0.002	0.174 ± 0.001	0.153 ± 0.000
Length deviation	6.72 ± 0.40	7.58 ± 0.42	11.67 ± 0.25
Unique rate (%)	97.76 ± 1.03	93.51 ± 1.39	83.89 ± 0.67
Novel rate (%)	100	100	100

Table S5. Accuracy comparison of peptide property prediction tasks on merged model. The table presents the property prediction accuracy across different models on the independent test sets. The metrics are reported for eight specific properties as well as the overall average performance.

Property	Galactica-6.7b	GPT-3.5-Turbo	Llama-2-7b	Ours
Overall	0.3621	0.5269 ± 0.0007	0.4716	0.9437
Assem	0.5392	0.6404 ± 0.0002	0.3592	0.9705
Amp	0.2029	0.1216 ± 0.0010	0.8888	0.9556
BBB	0.4737	0.3095 ± 0.0000	0.6585	0.8571
CPP	0.4576	0.3436 ± 0.0177	0.5538	0.8769
DPPIV	0.3700	0.3095 ± 0.0038	0.6899	0.7922
Hemo	0.9474	0.8933 ± 0.0000	0.1485	0.7697

Property	Galactica-6.7b	GPT-3.5-Turbo	Llama-2-7b	Ours
Soluble	0.4905	0.4903 ± 0.0007	0.5070	0.6930
Toxic	0.3145	0.3145 ± 0.0000	0.5000	0.7742

Table S6. Performance (ACC) of models trained solely on the property-prediction task. The table reports the prediction Accuracy (ACC) for each property task when the model is trained exclusively on the property-prediction subset (without instruction tuning or other datasets).

Model	assem	amp	BBB	CPP	DPPIV	hemo	soluble	toxic
single	0.9499	0.9739	0.7547	0.871	0.8647	0.8187	0.6522	0.7565

Table S7. Surrogate-estimated success rates for direct exploratory functional editing under varying sequence-similarity thresholds. The table presents the editing success rates for both predicted gain and predicted loss trajectories. The performance of the proposed model ('Ours') is compared against three baseline models under three sequence-similarity constraints: sequence identity > 30%, > 50%, and > 75%.

Similarity threshold	Task Direction	Property	GPT-3.5	Llama-2	Galactica	Ours
30	Gain	CPP	77.05	57.38	26.23	88.52
		BBB	75.86	43.1	44.83	91.38
		Amp	90	48.33	40	88.33
		Soluble	84.38	48.44	48.44	95.31
		Toxic	58.33	1.67	48.33	65
		Assem	59.7	28.36	32.84	77.61
		DPPIV	35.82	17.91	7.46	32.84
		Hemo	14.29	8.16	8.16	6.12
	Lose	CPP	2.99	0	4.48	4.48
		BBB	0	0	0	0
		Amp	3.03	0	3.03	4.55
		Soluble	0	0	0	0
		Toxic	24.29	18.57	17.14	20
		Assem	12.96	1.85	5.56	7.41
		DPPIV	60.81	22.97	24.32	66.22
		Hemo	77.42	19.35	46.77	79.03
50	Gain	CPP	72.13	26.23	13.11	88.52
		BBB	60.34	15.52	32.76	91.38
		Amp	83.33	43.33	20	81.67
		Soluble	75	48.44	20.31	92.19
		Toxic	50	1.67	16.67	61.67
		Assem	50.75	28.36	25.37	76.12
		DPPIV	32.84	2.99	5.97	32.84
		Hemo	12.24	4.08	6.12	6.12

Similarity threshold	Task Direction	Property	GPT-3.5	Llama-2	Galactica	Ours
	Lose	CPP	1.49	0	0	2.99
		BBB	0	0	0	0
		Amp	3.03	0	0	3.03
		Soluble	0	0	0	0
		Toxic	22.86	17.14	7.14	20
		Assem	11.11	1.85	1.85	7.41
		DPPIV	55.41	20.27	16.22	66.22
		Hemo	70.97	16.13	38.71	77.42
75	Gain	CPP	50.82	1.64	6.56	81.97
		BBB	48.28	3.45	27.59	86.21
		Amp	75	33.33	11.67	70
		Soluble	64.06	18.75	18.75	87.5
		Toxic	41.67	1.67	13.33	56.67
		Assem	38.81	19.4	14.93	70.15
		DPPIV	23.88	0	5.97	32.84
		Hemo	8.16	2.04	6.12	6.12
	Lose	CPP	1.49	0	0	1.49
		BBB	0	0	0	0
		Amp	3.03	0	0	3.03
		Soluble	0	0	0	0
		Toxic	20	14.29	5.71	17.14
		Assem	7.41	0	1.85	7.41
		DPPIV	50	16.22	12.16	60.81
		Hemo	61.29	9.68	37.1	70.97
